# When does life end? Consensus and controversy in defining death

**DOI:** 10.1007/s40592-025-00262-1

**Published:** 2025-08-02

**Authors:** Piotr Grzegorz Nowak

**Affiliations:** Institute of Philosophy, Jagiellonian University, Krakow, Poland

**Keywords:** Conflict of interest, Consensus, Brain death, Definition of death

## Abstract

After more than fifty years of debate on the definition of death, there remains no consensus among bioethicists. This article identifies the conflicting interests represented by various groups within the bioethics community as the primary cause of this stalemate. It argues that the impasse can be overcome if bioethicists recognize these conflicting interests as the fundamental reason for their disagreements, rather than viewing the dispute as primarily concerning the scientifically adequate concept of death. This article proposes a strategy on how to reach a consensus. The core idea in this regard is that the definition of death, in a socially important sense, needs to protect the interests of individual members of society.

## The three senses of life vs. death distinction

Throughout human history, severe respiratory failure has swiftly led to death, with few opportunities to use human organs to benefit others. However, the situation changed dramatically in the middle of the twentieth century. In Denmark, the ventilator was invented during the polio epidemic (Romero-Ávila et al., Revista Médica De Chile 148:822–830, 2020). Allowing clinicians to prevent the immediate progression from respiratory failure to organ deterioration. This technological advancement laid the groundwork for further medical breakthroughs. Subsequently, in the 1960s, Christiaan Barnard performed the first heart transplant (Singer, Rethinking life and death: The collapse of our traditional ethics, St. Martin’s Press, New York, 1996), revolutionizing organ transplantation. These advancements have raised profound Hamletian questions: ‘What does it mean to exist, and what does it mean to cease to exist? Where does the boundary between existence and nonexistence lie?’ Modern medical technologies challenge our traditional understanding of death, posing questions about whether our language—developed in a world without ventilators and organ transplants—is adequate to describe death in all its nuances. This dilemma is akin to pioneers from the tropics who are relocated to the Arctic; their language, with ‘snow’ as the sole term for all forms of frozen white water, limits their understanding of their new environment. As Alan Shewmon and Elisabeth Shemon suggest, these pioneers “would do well to learn the Eskimo [sic] vocabulary in order to focus attention on important aspects of their new reality, critical distinctions regarding that white stuff they so carelessly refer to as ‘snow’” (Shewmon and Shewmon, Brain Death and Disorders of Consciousness 2004:89–114, 2004). In the era before ventilators and advanced life-saving technologies, we were akin to pioneers living in the tropics, unacquainted with the complexities of the Arctic we now inhabit. Unlike those pioneers who could learn from the native Inuits (called “Eskimos” in the Shewmons’ original wording), we lack direct guides in our new environment and must redefine our language concerning life and death on our own. Bioethicists have been endeavoring to navigate this uncharted territory alone, without the help of ICU-specialized ‘Inuits’ (see for example Bernat et al., Annals of Internal Medicine 94:389–394, 1981; President’s Council on. 2008. Controversies in the determination of death. A White Paper of the President’s Council on Bioethics. Washington, DC.; Green and Wikler, Philosophy & Public Affairs 9:105–133, 1980; Lizza, The Journal of Medicine and Philosophy 18:351–374, 1993; McMahan, The ethics of killing: Problems at the margins of life, Oxford University Press, Oxford, 2002; Miller and Truog, Death, dying, and organ transplantation: Reconstructing medical ethics at the end of life, Oxford University Press, Oxford, 2012; Moschella, The Journal of Medicine and Philosophy 41:279–299, 2016a; Nair-Collins, Diametros 55:27–43, 2018a; Omelianchuk, The Journal of Medicine and Philosophy 46:530–560, 2021; Shewmon, The Journal of Medicine and Philosophy 26:457–478, 2001; Nowak, The Journal of Medicine and Philosophy: A Forum for Bioethics and Philosophy of Medicine 48:504–518, 2023). After more than fifty years of debate, is there an opportunity to reach a consensus among the disputants? In this article, I will propose a possible ground for such a consensus.

The first point on which perhaps all disputants can agree is that the life vs. death distinction, as commonly understood, is too nebulous to adequately capture all the shades of gray that appear in the modern ICU. For this reason, by building upon the rich debate, one can distinguish three distinct senses of this opposition.

First, by contrasting death with life, one might be contrasting inanimate objects such as rocks or skeletons with animated objects such as organisms, living parts of an organism, or the whole ecosystem of the earth. Let me call this sense of the herein-discussed distinction a simple one. Ervin Schrödinger quite well captured the essence of life in this regard in his famous book “What is Life?”:

When is a piece of matter said to be alive? When it goes on ‘doing something’, moving, exchanging material with its environment, and so forth, and that for a much longer period than we would expect an inanimate piece of matter to ‘keep going’ under similar circumstances. When a system that is not alive is isolated or placed in a uniform environment, all motion usually comes to a standstill very soon as a result of various kinds of friction; differences of electric or chemical potential are equalized, substances which tend to form a chemical compound do so, temperature becomes uniform by heat conduction (Schrodinger 2012).

Given this notion, an element is alive when there are some antientropic forces. Some forces are acting within the system, limiting the dynamism of energy dispersion in a stated temperature. It means nothing more that an element is alive when some forces within it restrain the dynamism of an increase in entropy. In this sense, all the following objects are live: a population of ants, a human organism, a kidney, an artificially sustained kidney, and a brain-dead patient supported by the ventilator.^1^ All these entities do not tend to thermal equilibrium in a way as inanimate objects such as a hot cup of tea do. I think when bioethicists like Michael Nair-Collins, Franklin Miller, and Robert Truog were pointing out that brain-dead patients are alive, they had this sense of life vs. death distinction (Miller and Truog 2012; Nair-Collins 2018a, 2024).

Second, by contrasting death with life, one might address the distinction between entities that are organisms and those that are no longer considered organisms—what can be termed as an ‘organismal sense’ of distinction. This aspect of the debate is complicated by a dominant perspective in the philosophy of biology known as organismal pluralism, which posits that no single definition of an ‘organism’ suits all biological contexts. Today, most biologists accept that the concept of an organism varies across different subdisciplines such as developmental biology, evolutionary biology, and physiology. Consequently, the question of whether an entity like a brain-dead patient is alive or dead as an organism requires specifying which subdiscipline of biology is relevant to the inquiry (Nowak and Stencel 2022; Stencel and Proszewska 2018; Nowak 2024b).

Finally, there is a sense in which when one contrasts life and death, one means the difference between beings that, in a sense, are still one of us, one of “the living,” and the entities that have ceased to be one of us (Green and Wikler 1980; Lizza 2011; Magnus 2018; McMahan 2002; Nowak 2023, 2024a; Veatch 2015; Shewmon and Shewmon 2004). Let us call this sense of distinction a social sense. If we are not just biological beings, some non-biological considerations, including social and ethical, might be crucial to grasping the nature of life and death here. What might be relevant in this context might be an analysis of the status of different entities in terms of their capacity to have interests (Nowak 2024b). If we are organisms, the third sense of distinction overlaps with the second one. But I do not think we are organisms, and as you will see in this article there are good reasons for you to agree with me (cf. McMahan 2002).

## What kind of definition have we been looking for?

The debate over the definition of death is a significant area of discussion in bioethics, with all participants aiming to establish a definition that could be adopted legally. Given that both law and bioethics are grounded in normativity, it is crucial for participants to recognize that defining death is not solely a biological endeavor. Instead, the sought-after definition should provide practical guidance for everyday life, ensuring it is both applicable and relevant in real-world scenarios.

I believe that all participants in the debate would agree that the status of a patient, in the simplest sense of the life vs. death distinction, does not hold primary importance for practical guidelines. For instance, the mere fact that an individual is ‘alive’ in this basic sense does not prevent their spouse from being considered a widow or widower (Veatch 2015). We can admit that a brain-dead patient is alive in the same sense as an artificially sustained kidney, and it does not preclude organ retrieval from them as long as the public is aware that they are alive in the simple sense of this word (Nair-Collins 2018b).

I also hope all can agree that there are different biologically valid concepts of organism. Some of them imply that brain-dead patients are functioning organisms, while others indicate that they are not any more organisms but something like cyborgs (Nowak 2024b; Nowak and Stencel 2022). Among all views on organism peculiar to natural sciences, there is no scientifically privileged perspective to adopt since no subdiscipline of biology is more accurate than another. In fact, if it is informed by theoretical biology, a choice of an organism concept is just that—it is a choice. It is dependent on people’s interests. In the following section, I want to consider the interests at stake.

## Different people and different interests

When biologists select among multiple biologically valid concepts of an organism, their choice is influenced by their specific scientific interests. For instance, an evolutionary biologist is likely to adopt an evolutionary perspective on organisms to clearly delineate the boundaries between individuals that participate in reproduction and contribute to the process of evolution by natural selection (Lewontin 1970). Conversely, if a researcher focuses on the development of various organs in animals or plants, they might prefer a developmental concept of an organism, which defines an organism as an entity progressing from ovum to ovum (Huxley 1854).

How about bioethicists who contribute to the definition-of-death debate? I can describe them as researchers who investigate the status of different ICU patients to provide practical guidance. Unlike natural scientists, bioethicists do not focus primarily on purely scientific questions, such as whether organisms in ICUs can participate in the process of evolution by natural selection or on the development (or deterioration) of different body parts. Instead, bioethicists typically champion non-scientific interests such as respect for autonomy, beneficence, the avoidance of harm, and equality (Beauchamp and Childress 2019). By examining how these interests are prioritized, I can identify three distinct groups of bioethicists engaged in the debate over the definition of death.

The first group, which I call the ‘pro-brain-death-pro-life’ camp, consists of those who believe that intentionally causing the death of an innocent human being is morally wrong. They also hold that life is sacred in a biological sense and adhere strongly to the principles of beneficence and nonmaleficence, advocating that individuals suffering from fatal organ failures should be helped, provided no harm is done to others. In their research, these bioethicists tend to adopt a functional developmental concept of an organism. According to this concept, an individual organism is a functionally integrated entity originating from a fertilized egg (Stencel and Proszewska 2018). This perspective not only supports their views but also serves their interests well by balancing the protection of biological life with the needs of organ recipients. It implies that a brain-dead patient, whose bodily integration relies on artificial support and not elements derived from a fertilized egg, is no longer considered an organism.

However, this group of researchers faces a dilemma. Their ethical framework, which upholds the prohibition of killing while also obligating the provision of benefits and the avoidance of harm, conflicts with the pure version of the functional developmental concept as presented in theoretical biology. Adhering strictly to this concept would be problematic, potentially causing offense and harm to many patients who, despite being conscious and sentient, rely on artificial support for survival.

To illustrate the concern, consider patients with pacemakers, which, like ventilators, do not originate from a fertilized egg yet are crucial for the patients’ biological integration (Nowak and Stencel 2022; Nowak 2024b). According to the strict functional developmental concept, this would imply that patients with pacemakers are similar to brain-dead patients, and are no longer organisms. Consequently, actions taken on such patients would not constitute killing in the traditional sense, as these patients would not be recognized as living organisms. This conclusion could lead to significant ethical and treatment dilemmas for many ill and disabled patients. To address these issues, the researchers from the first group advocate for modifications to the traditional functional developmental concept. Their goal is to maintain that while brain-dead patients may be considered dead as organisms, other sentient patients relying on artificial support, like those with pacemakers, should still be regarded as living organisms.

Of course, the categorization of bioethicists involved in the death definition debate as presented here is somewhat idealized. This means that, in reality, some bioethicists with the interests they advocate might not fit neatly into the proposed categories and could be placed somewhere in between. However, the clearest representatives of the first group include Moschella (2016a, 2016b, 2017, 2019), Condic (2016), and Bernat (2019).

Continuing this idealized division within the bioethicist community engaged in the death definition debate, the second group, which I term the ‘anti-brain-death-pro-life camp,’ shares interests with the first group but prioritizes them differently. For them, exercising extreme caution against intentional killings is more critical than facilitating organ transplantation. Like their counterparts, they hold that life, in some biological sense, is sacred. These researchers recognize that certain biologically valid concepts of an organism might classify brain-dead patients on ventilators as living organisms. Consequently, they oppose the retrieval of organs from such patients if it could result in death by any biologically valid definition, acknowledging the significant restrictions this places on organ transplantation. Notable bioethicists in this group include Shewmon (2001, 2010; Shewmon and Shewmon 2004), Nguyen (2016, 2018a, 2018b), Edmund Pellegrino (Bioethics 2008), Alfonso Gomez-Lobo (Bioethics 2008).

The third group of bioethicists in the debate primarily advocates for enhancing individuals’ capacity to make informed decisions. Contrary to the first and second groups, they do not believe it is inherently wrong for a physician to intentionally cause a patient’s death, provided there is informed consent and the act either does not harm the patient or only minimally harms them (Miller and Truog 2012; Nair-Collins 2018b; Truog and Robinson 2003). They are particularly concerned that both U.S. law and the laws of many other countries recognize brain death as the end of life, despite biologically valid concepts that might consider a brain-dead patient under artificial support as alive. Additionally, they point out that these patients are considered alive in a simple biological sense (cf. Nair-Collins 2024). This group, which I term the ‘anti-brain-death-pro-choice camp,’ believes it is crucial for the sake of personal autonomy that patients and their families are informed about the biological perspectives that recognize brain-dead patients as still alive.

While the overt interests of the three bioethicist camps are clear, some less visible motivations may also be at work. For example, certain members of both the second and third groups might favour limiting the transplant-surgery enterprise so that funds can be redirected to underfunded areas of health care. Torbjörn Tännsjö, for instance, argues for shifting resources from costly advanced surgical procedures—such as organ transplants—to bolster mental-health services (Tännsjö 2019).

When considering the choices of concepts of an organism by different bioethicists, it appears these decisions were traditionally made under the assumption of organismal monism—the belief that there is a single genuine concept of an organism and a singular true definition of death as the extinction of an individual organism (President’s Commision 1981; President’s Council 2008). Many bioethicists subscribing to some of the groups mentioned above were convinced that they were right, not their opponents coming from different camps. But perhaps something has changed recently. Possibly, participants in the debate are ready to accept the implications of organismal pluralism in the philosophy of biology. Maybe each of them is ready to admit that, in a sense, many of their opponents have been right. In some biologically valid sense brain-dead patients are dead as organisms. In some other biologically valid meaning, they are alive as organisms. Moreover, all bioethicists can agree that braindead patients are alive in the simple sense of the life vs. death dichotomy.

At the end of this section, I want to note an important reason that could perhaps explain why organismal pluralism with its consequences for the definition of death debate was overlooked to date. This reason has to do with a crucial difference between the interests pursued by biologists in their research and the interests pursued by medical ethicists in the bioethical debate on the definition of death. While the interests of biologists are usually not mutually exclusive, the interests of bioethicists in the definition of death debate often preclude one another. For example, suppose some biologists are interested in investigating the development of the excretory system in cats and adopt the functional developmental concept of an organism. In that case, such interest can be successfully pursued even if other biologists investigate the prevalence of some phenotypic trait in the population of cats and, therefore, adopt a version of an evolutionary concept. They identify cat organisms with slightly different physical objects, but this does not preclude their successful realization of their research goals.

The situation in the bioethical debate on the definition of death is markedly different. Here, some of the crucial interests advocated by bioethicists from divergent groups are mutually contradictory. For example, the interest in adopting a concept of an organism that maximizes organ retrieval directly conflicts with the interests of both camps of anti-brain-death bioethicists. Consequently, there exists a fundamental conflict of interest within bioethics, a phenomenon quite unusual among biologists defining borders of organisms. For this reason, it may be challenging for bioethicists aligned with different camps to acknowledge that no single group holds a monopoly on the correct concept of an organism, as such an acknowledgment could potentially undermine the interests they advocate.

## The proposal for a consensus

Suppose, however, that the time has come when bioethicists are ready to admit that there are different, equally valid biological concepts of an organism with varying verdicts on the status of brain-dead patients. Suppose they are also prepared to agree that brain-dead patients are alive in a simple, non-organismic sense. Moreover, suppose that now is the time when bioethicists can admit that the choice of a concept of an organism—though it must be informed by up-to-date theoretical biology to be correct—is, in fact, always significantly influenced by differing interests.

The interests that bioethicists have been advocating for are, in fact, not their own but those of diverse patient groups. Some bioethicists have primarily advocated for the interests of individuals on organ transplantation waiting lists. Others might, perhaps implicitly, have been advocating for the interests of people harmed by inadequate funding in certain areas of health care—for example, mental-health services. Additionally, some may have represented the interests of religious communities who believe that life, in any biological sense, is sacred and that intentionally causing one’s death in any sense is a sin.

If the community of bioethicists now recognizes how crucial interests are in choosing a definition of an organism and the associated definition of death, perhaps we are ready to more directly incorporate the consideration of different interests into the debate on the definition of death, moving beyond viewing death solely as the cessation of an organism. Bioethicists might be prepared for this shift if they acknowledge that some forms of human (or animal) existence, which retain the capacity to have interests, cannot be classified as organismal existence, since no subdiscipline of biology currently classifies such beings as organisms. These would be entities that remain sentient, yet all their functions identifiable as constitutive of an organism in any biologically valid sense are replaced by artificial machinery. Some participants in the debate, who constitute a fourth group of bioethicists, are already prepared for this shift, setting aside conceptual distinctions among divergent senses of “organism” (Veatch 2015; Veatch and Ross 2016; Lizza 2011; Green and Wikler 1980; McMahan 2002).

Many bioethicists regard impartiality as a hallmark of good practice. From this perspective, an ideal bioethicist cannot disregard the interests of non-organismal entities. Excluding certain interests solely because an individual does not meet the criteria of an organism would be akin to discriminatory practices such as racism or sexism—both of which are based on arbitrary biological distinctions like skin color. For this reason, I propose that all parties might agree that the central concern in the definition of death should be interests themselves, not merely biological factors. Since some interests cannot be adequately represented or safeguarded by any existing biological concept of an organism, and given that the statutory definition of death must hold practical significance in everyday life, it follows that death should be defined as a state in which an individual is permanently incapable of having any interests regardless how this interests are grounded by the physical and biological world.

Another argument that might persuade bioethicists to shift from considering a patient’s status solely as an organism to viewing it as an entity capable of having interests is the so-called ‘decapitation gambit’ (Lizza 2011). It is universally accepted that a decapitated body, in its current state, cannot have a stake in anything. Although it may still be associated with significant interests—perhaps the most critical ones—such as those of the person to whom the head once belonged, the body itself can no longer formulate, create, or retain any interests. If we assume that the head connected to this body has been destroyed, then the person whose body it was has died. Therefore, such an artificially sustained body should not be legally recognized as a living ‘we.’ It is not alive in a sense that is relevant to our everyday lives.

At the same time, many biologically valid perspectives, such as those from evolutionary biology, would suggest that such a body remains a living organism and is, in fact, the same organism as before decapitation, albeit severely injured (Nowak 2024b; Nowak and Stencel 2022). According to Ellen Clark’s evolutionary concept, the crucial factor is that these bodies preserve certain demarcation and policing mechanisms (Clarke 2010, 2013). Specifically, the body continues to maintain a distinction between the production of somatic and germ-line cells. This distinction enhances the likelihood of the organism’s reproduction, more so than if such cellular differentiation were absent. Additionally, the body retains mechanisms for preprogrammed cell death and possesses a functioning immune system that prevents individual parts of the organism from reproducing independently. In essence, a decapitated corpus remains a unit capable of potentially reproducing itself and participating in the evolutionary process by natural selection.

As I mentioned at the beginning, the language about death that we have inherited is not precise enough to capture all the death-related phenomena that appear in ICU units. Discussing life and death in the ICU is straightforward when we refer to the simple sense of the distinction. However, applying this distinction in an organismal sense presents much greater challenges. Both interpretations of the distinction correspond to natural transitions that occur in the real world. Yet, as noted earlier, there is also a third aspect of being alive, one based on interests, which throughout the history of our language has become intertwined with the other senses.

In this sense, if someone or something is alive, there are ways to benefit or harm this particular being as it is. If there was no such a sense of life vs. death distinction, statements such as that “death is a tragedy” (Perhaps because, as Thomas Nagel says, “it brings to an end all the goods that life contain” (1970)) would not be so deeply rooted in our cultures.

Throughout history, the putrefaction of a body typically followed the extinction of an organism. Similarly, the cessation of an organism usually coincided with a point when no new interests could be developed. Previously, there was no need to distinguish among the three senses of the life vs. death distinction. However, the advancement of artificial medical technologies has demonstrated that the capacity to have interests and the life of an organism do not always coincide. There might be individuals who cannot be classified as organisms in any biologically valid sense yet remain conscious and capable of forming desires (Nowak 2023, 2024b). Furthermore, as highlighted by the decapitation gambit, there is no inherent reason to assume that sentience or any sophisticated vulnerability to harm and benefit necessarily persists as long as the organism exists (Lizza 2011).

The fact that the third sense of life vs. death distinction goes apart from simple and organismal sense does not make the criteria for being an entity that might be benefited or harmed less real or physically ungrounded than the criteria for being an organism or being an animated part of this world. Our vulnerabilities to harm and potential for benefits are just as rooted in the physical world as our organisms’ capacity to maintain homeostasis. Benefits cannot be received nor harm inflicted if one lacks interests. Interests cease to exist without the capacity for affective states,^2^ which expires along with our brains. Without the capacity for affective states, one cannot experience pain or pleasure, nor form preferences or desires, or engage in higher-level approval or disapproval of these feelings or desires (Nowak 2024a). Consequently, a decapitated corpus under artificial support, lacking any interests, cannot benefit from or be harmed by anything. Given this third aspect of the distinction, such a body is, therefore, dead, and this death is no less actual than the death of an organism or a piece of tissue. To claim that such a decapitated corpus is alive in this third sense amounts to creating fiction. This fiction, or to frame it differently, such a ‘noble lie,’ might be justified in certain circumstances, for example, to respect the religious or moral beliefs of those who adhere to the doctrine of the sanctity of life and concurrently believe that life in the simple or organismal sense is ‘sacred’ (cf. Miller and Truog 2012).

At the end of this section, let me make three further notices. First, note that the recognition that death in a third sense comes with irreversible cessation of affective states does not preclude the possibility of posthumous harm. Moreover, the view which holds that there is such a thing as posthumous harm does not imply that one can survive the destruction of one’s brain. To see this, let me clarify what it means that one is capable of having interests.

First, when one is braindead, one cannot experience anything and will never regain the possibility of experiencing anything. Bare sentience, which is feeling pain or pleasure, is the most basic way of having an affective attitude and showing interest. When brain-dead, one cannot be interested in anything in this primary sense.

Capacity for sentience is a way of showing an interest common for humans and many animals. Yet there are also different, more sophisticated ways of showing an interest, which are, in a way, built upon sentience. Consider the capacity to have desires or, as some say, preferences. Following Chris Heathwood, I define the capacity to have desires as a capacity to be genuinely attracted with the perspective that something can happen or could happen (Heathwood 2019). As you can see, the capacity to have desires has two aspects. First, one who has this capacity is capable of conceiving alternative possibilities taking place in the world. Second, one can also be genuinely attracted to a different extent by different alternatives. This second “attractive” aspect of the capacity to have desires reveals that this capacity is a sophisticated type of sentience. The brain-dead patient obviously cannot be “attracted” by anything, nor can they conceive anything. Therefore, they obviously cannot create any new desires. However, before lapsing into brain death, they probably had wishes regarding how their braindead body should be treated. These desires might still be morally binding. Whether this is the case is beyond the scope of this article.

The capacity to have desires is a capacity to show interest, which might be inaccessible to many animals, but some, such as great apes, might have it just as humans (Regan 2004). Finally, there is also the most sophisticated capacity level for having interests. Let me name it “practical reflection” (Frankfurt 1988; Korsgaard 1996, 2009, 2018; Nowak 2024a; Regan 2004). Namely, one can be interested in getting rid of some of their desires, or one can be interested in experiencing some pain. Here is an example: a pedophile might want to get rid of the desire to have sex with a child. They might also revolt against experiencing the pleasure of having sex with a child and want to feel some pain or suffering instead. This third kind of capacity to have interests can be described as having sensational (affective) attitudes towards one’s desires and bare sensations. It is the most sophisticated way of having interests. Just as with desires, this dimension of capacity for creating new interests is gone with brain death, although it might be that the person before becoming braindead had undergone some practical reflection on fulfilling some of their first-level desires after their death. The fact that such practical reflection was conducted might still be morally binding for how we treat the braindead body. I want to stay neutral on whether it is, in fact, ethically imperative. In the following paragraphs, I will show that if posthumous harm exists and is morally imperative, it is compatible with recognizing brain death as a death in the sense of permanent cessation of capacity to have interests.

As I have already said, after the destruction of the brain, no affective states are possible anymore. No pains or pleasures can influence one’s fate after brain death. Yet some believe that posthumous harm is something that might happen. The possibility of posthumous harm results from the fact that most human beings, before the destruction of their brains, were able to create desires. In particular, these wishes could be about things that happen to their bodies after their brains are destroyed.

Some believe that following such wishes benefits human beings posthumously. If one believes in posthumous harm, one upholds that the deceased person is harmed whenever their wish regarding the fate of their remaining body or any other objects or events becomes frustrated. Importantly, it is not a braindead body that is harmed, nor is it a putrefied body (Nowak 2018). According to those who believe in posthumous harm, the harmed subject is the person who existed in the past (Feinberg 1987). Therefore, posthumous harm is compatible with brain death being death. Yet, one of the problems associated with posthumous harm is that it seems to assume backward causation (Portmore 2007; Pitcher 1984). Some argue that this is a fatal problem and, therefore, reject the possibility of posthumous harm. However, I want to remain neutral about this issue in this article. What is essential for my purpose is that death as a permanent incapacity to create interests is compatible with moral theories, which assume the existence of such a thing as posthumous harm, and with the theories that deny such a possibility.

I take the neutrality of this consensus proposal on the issue of posthumous harm to be an advantage. Moreover, what I also believe to be an advantage of this stance on the definition of death is that the view I propose remains neutral on controversial metaethical issues. On the one hand, the view can be adopted by those who believe there are no moral obligations or rights, including individuals who regard discussions of moral obligations and rights as erroneous, or those who see such discussions as merely expressive of noncognitive mental states like emotions or desires (Mackie 1977; Stevenson 1937). On the other hand, the same view can also be embraced by moral realists who affirm the existence of moral rights and duties (Nowak 2024a).

The view is also neutral when choosing a theory of normative ethics. One can be a utilitarian, Kantian, virtue theorist, divine commands theoretic, or whoever one wants to be regarding normative ethics. One can believe that putting an end to the existence of animated material is wrong even though such living biological material per se is not capable of having any interests based on affective states. Note that this is an example of an extreme biocentric view in the style of Schweizer (1979). To take the opposite view, one could believe that it is morally wrong to sustain vital functions in end-stage dementia patients (cf. Velleman 1999). The view remains neutral on whether these or any other moral views are correct or incorrect. It only says that death in the relevant sense of everyday life and medical practice is a matter of capacity for having interests that are based on affective states. It is a state in which one cannot be harmed or benefited besides the very peculiar possibility of posthumous harm (provided that posthumous harm is possible).

The proposed consensus itself does not make any assumptions regarding whether there is a moral obligation to help people or to avoid harming them. In particular, the view does not imply that if there is such an obligation, it must be based on people’s interests. Suppose one would make such a moral claim. In that case, one can take, for example, the side of utilitarians in normative ethics, the side of interest-based moral rights view (see, e.g., Joseph 1986), or one of many other moral views that are compatible with the belief that interests can ground moral obligations. However, the proposal itself does not force anyone to be a utilitarian, interest-based moral rights theory proponent or to any particular stance in normative ethics. Someone who accepts it might still believe utilitarians are wrong about morality, and there are deontological constraints like divine commands that are not based on the capacity to have interests (Adams 1987). The proponent of divine command theory, or some other theory that assumes that moral obligations are not grounded in any interests, can remain on the board as long as such a person accepts that death in a practically relevant sense is a permanent loss of capacity to have interests.

## Practical implications of the consensus proposal

To summarize the theoretical part of the article, the key claim is that death, in the third sense—relevant for medical practice—is the permanent cessation of the capacity to have interests. Having demonstrated my justification for remaining neutral on controversies in normative ethics, I will now focus on the practical consequences of the consensus proposal for defining death in this final section.

Let me start with what is most apparent: brain death, a state from which there is no return neither to sentient nor conscious life, remains a valid criterion for death. Can anyone be sure that the tests for brain death are perfect and give no false positive results? To date, no one has proved beyond a doubt that in any case of diagnosis of brain death, consciousness returned. There is, however, one contested case of Jahi McMath (Shewmon 2018a, 2018b; Shewmon and Salamon 2021). Jahi was diagnosed as braindead in 2013 and a few years later regained minimal consciousness, according to Alan Shewmon. In Shewmon’s interpretation, the case of Jahi is not a case when one recovers from brain death but rather a case of a false diagnosis. According to him, Jahi was never brain-dead. Instead, she suffered global ischemic penumbra with little blood flow to the brain. There was so little flow that, due to technical obstacles, it was impossible to detect it. However, the flow was sufficient to heal Jahi’s brain to the extent compatible with minimal consciousness.

Shewmon’s assertion that Jahi had progressed to a minimally conscious state is based on an analysis of videos taken by Jahi’s family, which depict her alleged conscious responses to commands such as ‘give us a thumb up,’ and on a single examination where he reportedly observed Jahi’s intentional response to similar simple commands. However, such evidence is not considered scientifically valid. Shewmon’s hypothesis has been criticized by experts in the field (Machado 2021; Lewis 2018).

What is worth noticing in the context of Jahi’s case is that to the date, no prospective study has followed brain-dead patients on continued life support long enough to establish a definitive false-positive rate; most published work instead compares ancillary tests with the standard bedside examination (see for exeample: Chassé et al. 2025) or reports isolated cases of diagnostic error. A unique study in this regard is Alan Shewmon’s meta-analysis of the prolonged survival of organismic functioning cases in brain-dead patients (Shewmon 1998). It is important to observe that in none of the cases of what Shewmon termed “chronic brain death,” there were any signs of regaining consciousness in patients. However, as Shewmon says, the diagnosis of brain death is usually a self-fulfilling prophecy in the sense that in the event of this diagnosis, either the ventilator is withdrawn or organs are procured, and for this reason, the brain-dead patient usually becomes quickly dead also in the biologically simple sense. Since all diagnostic procedures are somewhat defective, what is needed is an appropriate study to determine if there is any rate of false-positive diagnosis in case of brain death. What is crucial is to investigate whether, according to Shewmon’s hypothesis, global ischemic penumbra can mimic brain death and if it is possible for patients who suffer from global ischemic penumbra mimicking brain death to regain minimal consciousness.

With the minimal evidence available, including Shewmon’s meta-analysis and the single case of Jahi McMath, we can only speculate on the possible outcomes of a more comprehensive study. Suppose Jahi McMath did regain minimal consciousness and was misdiagnosed, not actually brain-dead but suffering from a global ischemic penumbra. Meanwhile, none of the 56 patients in Shewmon’s chronic brain death meta-analysis regained consciousness. Assuming this sample of 57 patients reflects the broader population diagnosed with brain death, we might estimate a false positive diagnosis rate of 1.75%. This figure likely overstates the error rate since the sample is not representative of all individuals diagnosed as brain-dead; it might, at best, represent those whose bodies can be maintained over extended periods. However, if 1.75% of brain-dead diagnosed patients could potentially regain minimal consciousness, we must question whether this error rate is acceptable.

In medicine, almost no diagnosis is entirely beyond doubt. This holds true for the diagnosis of brain death, which, like any medical determination, is not immune to error. Similarly, diagnoses based on cardiological criteria are fallible; there are media reports of patients pronounced dead by these criteria who later regain consciousness in morgues (Dockterman 2014). The only death criterion considered significantly more reliable involves visible signs of putrefaction, such as rigor mortis (Oderberg 2019). Yet, even here, errors can occur, as demonstrated in 2018 when Gonzalo Montoya Jiménez was mistakenly declared dead due to signs that were misinterpreted as rigor mortis but were actually catalepsy (Dockrill 2021; cf. McGee and Gardiner 2017).

Moreover, as a society, we cannot agree to adopt rigor mortis as a default criterion of death, even though it is a more certain criterion than the neurological or circulatory-respiratory ones. If we adopt such a criterion, everyone in society would be worse off because significant amounts of scarce medical resources, such as ventilators, and the time of ICU staff and other medical staff would be used to treat patients who have no brain or circulatory functions but do not yet show signs of body putrefaction. What we can and probably should do is to assess how likely it is that a diagnosis of death, either by neurologic or cardiac criteria, is mistaken in a way that is ethically and socially significant—namely, that the misdiagnosed patient could regain consciousness.

Someone could object to what I say here, upholding that only rigor mortis is an appropriate criterion of death. Defending rigor mortis as the best criterion one could point out that even though death should not be determined based on circulatory criteria nor based on neurological criteria, due to the fallacy of these, we should determine that treatment of patients who are positively diagnosed for brain death or who fulfill circulatory criteria for death are hopelessly ill and their further treatment should be judged to be futile and for this reason stopped (cf. Bioethics 2008).

I do not think such an objection undermines the proposed herein view on death. This is because the view on death presented in this article is akin to a specific type of judgment about futility. Joanne Lynn and James F. Childress distinguished three types of futility, one of which was the lack of the possibility of being harmed or benefited (1983). This type of futility is implicit in the understanding of death that this article proposes. The difference is that the judgment on futility as such is a normative *prescription*, while the determination of death is the *description* of a fact. When it comes to treating patients who are beyond harm and benefits, Lynn and Childress’ point is that *it is allowed* to withdraw or withhold their treatment. This is a moral issue. However, the determination of death per se is not a formulation of a moral judgment, even though such judgment, namely that one is *allowed* to stop treatment of a dead person, can be easily made when death is determined.

Note that there is a critical difference between the futility of treatment for patients who are beyond harm and benefit, and the more common cases where therapy is deemed disproportionately burdensome (Lynn and Childress 1983). The latter typically involves patients who are either currently conscious and sentient, or who have the potential to regain consciousness and sentience. Determining whether treatment is disproportionately burdensome relies on accurately assessing the patient’s interests. In cases where the patient is incompetent, surrogate decision-makers and physicians must collaboratively assess the benefits and harms of treatment options as the patient would if competent. If consensus is lacking, ethical committees may be consulted, and controversies might ultimately be resolved in court (Truog and Mitchell 2006; Beauchamp and Childress 2019). This decision-making process is justified when patients have not irretrievably lost their capacity to generate interests, being either presently able or potentially able to value or disvalue their conditions.

However, applying the same decision-making process for futile treatment to patients who have no circulatory functions for a prolonged time or are brain-dead would be a mistake. These patients are beyond the possibility of harm and benefit. It is senseless to question whether their current state is burdensome for them, as brain-dead bodies or corpses without circulation cannot be subjected to such states. The only conceivable harm that might affect these patients is posthumous harm. How much moral weight should posthumous harm carry? Addressing this ethical question demands substantive moral discussion. Nonetheless, it should be recognized that posthumous harm generally carries less significance than harm to living patients. In practice, this means extending treatment to brain-dead patients should only be considered if it incurs no cost to living patients.

In this final section, I also want to address the practical issue of consent in determining death by neurological criteria. When a patient is admitted to the hospital, treatment and diagnosis typically require some form of consent. For many planned diagnostics and therapeutic procedures, patients provide explicit informed consent after being informed about the nature of the medical process, its risks, alternatives, and so forth. However, certain procedures are performed without patients individually authorizing them through explicit consent. For instance, nurses may measure a patient’s body temperature at night without awakening them. It is understood that patients implicitly consent to such procedures by virtue of being admitted to the hospital and not expressing a desire to leave (Beauchamp and Childress 2019).

Is brain death testing akin to bodily temperature measurement? The examination for brain death is undeniably more intricate. For instance, consider a blood flow study, which may be part of the tests for brain death (Greer et al. 2023). Typically, if such a study is conducted in the hospital for reasons other than diagnosing brain death, explicit informed consent for the procedure is usually required from the patient or their surrogate. Secondly, unlike many diagnostic procedures aimed at improving patient health, determining death by neurological criteria does not serve to enhance the patient’s well-being. Moreover, if the patient is not actually brain dead, such a procedure may entail some level of risk for them (Miller and Truog 2012; Berkowitz and Garrett 2020).

The most contentious element of the brain death examination is the apnea test. Coimbra suggests that the apnea test could potentially harm a patient’s brain by elevating intracranial pressure (1999). Despite the lack of empirical confirmation regarding its detrimental effects, the theoretical possibility, the absence of immediate benefits, and the standard practice of obtaining distinct consent for similarly complex procedures warrant the consideration of obtaining consent from patients undergoing determination of death by neurological criteria.

Suppose that we adopt a policy of retrieving consent for brain death testing. Now consider the case in which the surrogates do not consent to determine death by neurological criteria, with its consequence of treatment discontinuation. Given that the physicians suspect the patient to be brain dead, their treatment should be anyway judged to be futile within some futility protocol. However, if surrogates disagree with brain death testing with its consequence of treatment discontinuation, they will probably disagree also with physicians’ opinion that further treatment is futile and the life-sustaining treatment should be withheld. Should physicians then wait for a court resolution? Suppose the answer to this question is yes, and we adopt the policy of retrieving consent to determine brain death. In that case, physicians may be reluctant to initiate mechanical ventilation when they perceive a significant risk that continued support would prove medically futile, or when they anticipate that the patient (or surrogate) will refuse neurologic determination of death. Alternatively, we might find ourselves in a situation where there will be a substantial number of brain-dead patients at ICUs whose families do not agree to a diagnosis of brain death and who also do not agree to discontinue life-sustaining treatment. Such a consequence might be acceptable as long as the number of brain-dead patients on artificial support would not deprive any living patient of getting access to care at the ICU.

Contrarily, if there is a risk that living patients might be deprived of care due to allocating scarce resources to the brain-dead, we must address these ethical dilemmas through the lens of the ICU’s inherent morality (cf. Miller and Brody 2001). Critical resources such as ventilators, ECMOs, feeding tubes, and skilled personnel are significantly scarcer than common medications like paracetamol. ICUs can uphold the best interests of society by judiciously using these limited resources. Typically, treatments for patients diagnosed with brain death are discontinued, which allows ICUs to treat others who have prospects for recovery. This policy inadvertently benefits even those whose families oppose a brain death diagnosis. They “owe” the possibility of their resistance to the treatment discontinuation policy, which facilitated their ICU admission. Without such policies, some patients might never access ICU care and could die earlier due to the lack of ventilator support. Thus, it becomes evident that some opponents of treatment discontinuation were, in fact, past beneficiaries of the very policy they contest. Consequently, when the well-being of living patients is compromised by decisions made on behalf of brain-dead patients, the needs of the living must be prioritized. Nonetheless, it is crucial to inform the public that in ICUs, where artificial life support is extensively utilized, death is often determined neurologically. Naturally, patients should not be compelled to remain in the ICU against their will, nor should the continuation of treatment for brain-dead patients occur if it jeopardizes the care of living patients.

## Conclusions

This article has sought to establish a foundation for consensus on the definition of death. You may rightly wonder, ‘What are the chances that this proposal will persuade the majority of experts after more than fifty years of debate?’ If you are skeptical, you likely assume the chances are low. While I am not a skeptic, I am also not overly optimistic about the immediate possibility of reaching a consensus. However, I envision this article as providing a framework that might guide experts toward achieving actual consensus in the future.

First, if consensus is to be achieved, experts should identify the sense in which they have been working on the definition of death and specify whether it was the simple sense, the organismal sense, or the social one. This operation might reveal that the disagreement among some of the experts was superficial, as they were addressing different senses of death. If the experts can agree that there are three senses of death, as identified in my article, they should also be able to agree that brain-dead patients are alive in the simple sense, dead or alive in the organismal sense (depending on the choice of a biologically valid concept of an organism), and dead in the social sense. To reach such an agreement, experts must accept the assumptions of organismal pluralism in theoretical biology. They also need to assume that the social sense of death does not overlap with the simple-biological or organismal-biological senses, and that it is a matter of capacity to have interests.

There might be impediments to fulfilling these conditions. For example, some participants in the debate might insist, contrary to the mainstream stance in theoretical biology, that there is one privileged concept of an organism and that the organismal sense of the life vs. death distinction overlaps with the socially important sense of the distinction. Moreover, they may argue that it is not interests that matter, but whether one is a (human) organism. Others might take a different position, maintaining that neither the simple biological sense nor the organismal sense matters; however, they believe that there is no privileged sense of death in a social sense, and that each person can have their own valid definition of death in the social sense, applicable to that particular person.

Both of these stances, which stand in the way of consensus, present significant challenges and represent extreme positions on opposite ends of the spectrum. Those who insist that a privileged organismal sense of death aligns with the social sense must contend with the mainstream position in theoretical biology, an undertaking that is highly ambitious. Additionally, their approach is problematic because it overlooks the importance of individual interests in favor of prioritizing the biological categorization of being an organism. It is difficult to understand the rationale behind disregarding the diverse interests of people, asserting instead that the mere fact of being an organism is what truly matters.

At the other end of the spectrum, there are participants in the debate arguing that it is dogmatic to define the boundary between life and death in a social sense to be based solely on the capacity to have interests. These disputants maintain that individuals should have the freedom to define their own death in a social sense, without any imposed constraints.

This extreme viewpoint presents several problems. First, it leads to chaos (cf. Williams 2017). Consider a society where some people define their death in a social sense as the onset of the first symptoms of dementia. Meanwhile, others, like the Torajans, believe that death occurs many weeks after the cessation of both circulation and brain functions (Budiman 2014). Additionally, there are individuals who have not specified their own concept of social death. How, then, should the death of these individuals be pronounced?

We cannot allow such chaos in the determination of death policy. In a liberal democratic society, we protect people’s interests irrespective of their race, gender, or sexual orientation (Raz 2004). Consider the Torajans, who may believe they are alive in the socially important sense weeks after their heart and brain have ceased functioning, and may even wish to be fed during this period (Budiman 2014). If honoring the wishes of the Torajans holds any moral significance, it stems from these individuals’ capacity to form desires, preferences, and plans, and to experience suffering and satisfaction while they were alive. The practice of feeding decaying bodies, as per Torajan beliefs, is not justified by any metaphysical or physical properties of these bodies. Instead, the importance of treating Torajans according to their religious beliefs originates from their ability to formulate interests throughout most of their lives. In essence, it is the full range and diversity of these interests that confer significance. Without the capacity to have interests, an individual cannot impact what is socially significant and is considered dead, irrespective of whether they are Torajan, American, Polish, or belong to any other cultural group.

I hope that the obstacles to achieving a consensus can be overcome by dismissing the extreme positions. This may be feasible, particularly since the strategy proposed here acknowledges the theoretical and ethical merits of the definitions of death advocated by representatives from all camps throughout the debate. Experts from the ‘pro-brain-death-pro-life’ camp are commended for developing a concept of an organism that not only serves the interests of transplant recipients but also aligns organ retrieval from brain-dead patients with the doctrine of the sanctity of life and the traditional prohibition against killing. Experts from the ‘anti-brain-death-pro-life’ camp are recognized for highlighting that in some biologically valid organismal senses, brain-dead patients on artificial support are alive, thereby safeguarding the interests of those for whom the traditional prohibition of killing is paramount, urging extreme caution. Lastly, the ‘anti-brain-death-pro-choice’ camp is valued for promoting human autonomy and the significance of informed consent by emphasizing that individuals making end-of-life decisions should understand that brain death is a state where a patient is still alive in the simple biological sense.

All parties involved in the debate have grasped at least a part of the truth concerning the nature of death and have employed effective means to protect the interests of the people they implicitly represent. To achieve consensus, it is crucial for them to mutually recognize the merits of each other’s positions and acknowledge that the interests they champion are, to some extent, contradictory. Moreover, reaching a consensus requires a willingness among experts to engage in collaborative efforts. However, it is not guaranteed that such a willingness exists. Once again, conflicting interests may pose a barrier. This is compounded by the personal interests of bioethicists, who often work in academic environments that foster disagreement to encourage innovative and original research. Such environments may inadvertently promote dissent among experts, as novel academic contributions in fields like theoretical bioethics frequently challenge the status quo rather than seek to establish or support it. Consequently, the structural dynamics of academia may inherently discourage consensus on theoretical matters such as the definition of death.

## Data Availability

No datasets were generated or analysed during the current study.

## Footnotes

1 Note that Schrödinger’s remark that “when a system that is not alive is isolated (…) motion comes to a standstill” refers to inanimate matter; the living system, by contrast, must remain open, continuously exchanging energy or material—whether through spontaneous physiology or technologically assisted means such as a ventilator.

2 In this article by affective state I understand any valenced, experience-laden or motivational mental state such as pain, pleasure, emotions, desires and higher-order approvals or disapprovals.
